# Computer-Aided Design of 3D-Printed Clay-Based Composite Mortars Reinforced with Bioinspired Lattice Structures

**DOI:** 10.3390/biomimetics9070424

**Published:** 2024-07-11

**Authors:** Nikolaos Kladovasilakis, Sotirios Pemas, Eleftheria Maria Pechlivani

**Affiliations:** Centre for Research and Technology Hellas, Information Technologies Institute, 6th km Charilaou-Thermi Road, 57001 Thessaloniki, Greece; sopemas@iti.gr (S.P.); riapechl@iti.gr (E.M.P.)

**Keywords:** conceptual design, biomimicry, lattice structures, 3D printed clay-based composite, computation mechanics, non-cementitious materials

## Abstract

Towards a sustainable future in construction, worldwide efforts aim to reduce cement use as a binder core material in concrete, addressing production costs, environmental concerns, and circular economy criteria. In the last decade, numerous studies have explored cement substitutes (e.g., fly ash, silica fume, clay-based materials, etc.) and methods to mimic the mechanical performance of cement by integrating polymeric meshes into their matrix. In this study, a systemic approach incorporating computer aid and biomimetics is utilized for the development of 3D-printed clay-based composite mortar reinforced with advanced polymeric bioinspired lattice structures, such as honeycombs and Voronoi patterns. These natural lattices were designed and integrated into the 3D-printed clay-based prisms. Then, these configurations were numerically examined as bioinspired lattice applications under three-point bending and realistic loading conditions, and proper Finite Element Models (FEMs) were developed. The extracted mechanical responses were observed, and a conceptual redesign of the bioinspired lattice structures was conducted to mitigate high-stress concentration regions and optimize the structures’ overall mechanical performance. The optimized bioinspired lattice structures were also examined under the same conditions to verify their mechanical superiority. The results showed that the clay-based prism with honeycomb reinforcement revealed superior mechanical performance compared to the other and is a suitable candidate for further research. The outcomes of this study intend to further research into non-cementitious materials suitable for industrial and civil applications.

## 1. Introduction

Additive manufacturing (AM) is becoming increasingly popular and proving its benefits across various sectors [1,2,3,4]. Due to its ability to create complex geometric shapes, AM is becoming a game-changing technology in the construction industry [5,6,7,8]. The advantages it offers are numerous, including reducing labor accidents, construction time, and total costs, and providing construction freedom, making its adoption inevitable [9,10,11]. Additionally, AM enables eco-friendly construction methodologies, which are crucial [12,13]. To date, new 3D printing systems with multiple innovations have emerged, offering reliable and innovative construction solutions for both small structures like kiosks, bridges, and benches, and larger-scale projects such as residences and shops [14,15,16,17,18]. These innovations are also vital for quick building repairs and creating temporary housing in disaster situations like earthquakes [19,20,21,22].

The AM technology used for building purposes can be found in the literature referred to as 3D mortar printing (3DMP), 3D concrete printing (3DCP), and liquid deposition modeling (LDM) [23,24,25,26]. All these methodologies share the use of a liquid or paste-like mixture suitable for building purposes. By applying this mixture with layer-by-layer extrusion, the desired geometry is achieved [27,28]. A less developed method in 3D concrete printing is shotcrete 3D printing, which utilizes a shotcrete spraying nozzle mounted on an articulated robotic arm to apply concrete and construct a structure based on a digital design. This method addresses weak interlayer bonding by spraying concrete at high pressure over the previous layer, requiring an optimal time window where the concrete is firm enough to resist distortion but still able to bond with the adjacent layer [29,30].

Such technologies offer potential for mass customization and rapid manufacturing but face challenges like weak interlayer bonding and anisotropic material behavior due to layer-by-layer deposition [31]. To mitigate these weaknesses and improve the mechanical performance of the entire construction, solutions must be encountered. According to the literature, a very effective enhancement can be achieved by integrating supportive structures like meshes or fibers [32,33].

Nowadays, several studies have examined various approaches for using reinforcing meshes in cementitious and earthen mixtures extruded by additive manufacturing techniques, with the most commonly used methods being in-layer reinforcement and across-the-layer reinforcement [31,33,34,35,36]. Liu et al. [33] applied reinforcement meshes in 3D concrete printing using 304 stainless steel mesh with a grid size of 6.0 mm × 6.0 mm and a wire diameter of 0.6 mm on top of the printed layers. Destructive and non-destructive tests show that these reinforcement meshes change failure modes from brittle to ductile, validating that this reinforcement method enhances strength and toughness. Kristombu Baduge et al. [31] emphasized the importance of reinforcing 3DCP structural elements to increase their flexural, shear, and tensile capacity. They noted that longitudinal and lateral reinforcement improves ductility, crucial for earthquake resistance. They mentioned reinforcing techniques such as pre- and post-installed reinforcement methods commonly used for large-scale 3DCP structures, involving placing reinforcement before or after printing. Moreover, as another reinforcement method, they presented the fiber reinforcement method, which integrates fibers inside concrete or mortar to increase strength. Fibers can be added manually, mixed with the printing mixture, or extruded with customized machines to avoid clogging. Regarding the across-the-layer reinforcement method, Marchment et al. [34], introduced an in-process method to embed mesh reinforcement. A custom nozzle was employed, allowing mesh reinforcement to be overlapped simultaneously with concrete layer printing, achieving continuous reinforcement. The results show that strong bonds between the mesh and material are created. Specifically, steel reinforcement increased moment strength in flexure by 170–290%, and bond strength was 42% of the cementitious mix’s tensile strength. Furthermore, Liu et al. [37], propose a novel reinforcing approach for 3DCP via U-type steel wire mesh to provide both integrated horizontal and vertical reinforcements. Flexural tests demonstrate that printed specimens with U-type steel wire mesh exhibit a significantly higher load-bearing capacity and deflection hardening compared to those with flat steel wire mesh and those without reinforcement.

The present study investigates an innovative approach to improving construction materials with low or zero cement content, aiming to replace traditional cement-based materials. To do so, the proposed methodology for enhancing mechanical performance involves integrating polymeric meshes, especially with biomimetic designs, into clay-based 3D-printed prisms. Specifically, in simulations with the 3D-printed prisms, three different designs of polymeric lattice structures are selected and examined for their reinforcing capabilities. These designs include a commonly used cubic grid structure in construction applications, and two bioinspired structures: honeycombs and Voronoi patterns. These three designs are tested in three-point bending using an explicit dynamics FEA based on the ANSYS code. The materials composing the reinforced prism are a mixture of clay (1 kg), water (0.375 kg), ceramic powder (0.2 kg), and plasticizer (0.02 kg). The reinforcing meshes are made of poly(lactic acid) (PLA), selected for its ease of printing and because it is one of the most commonly used polymers in 3D printing applications [38,39,40]. Moreover, the calculated FEA models pertain to prisms fabricated using LDM technology, with integrated meshes produced through FFF technology. The scope of this study is to develop models so that more meshes with different patterns and dimensions can be examined for construction applications using 3D printing technologies.

**Figure 1:**
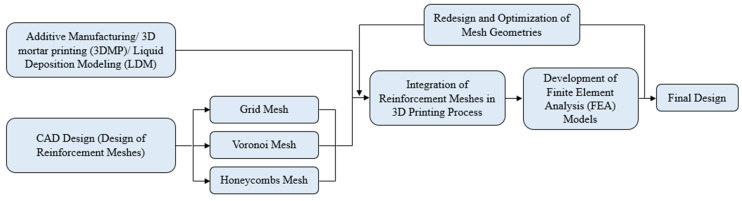
Flowchart of reinforcement mesh selection procedure.

The current study’s structure is briefly demonstrated in Figure 1. This figure portrays a flowchart of the present study and essentially describes the methodology followed for developing finite element analysis (FEA) models for examining various cases of reinforcing meshes in clay-based mortars. Section 2 of this study presents the materials and methods used for the investigation. Section 3 showcases the results obtained from the FEA simulations. Finally, the study concludes in Section 4 with overall outcomes and suggestions for further examination of different cases.

## 2. Materials and Methods

### 2.1. 3D Printing and Mechanical Properties of Clay-Based Specimens

**Figure 2:**
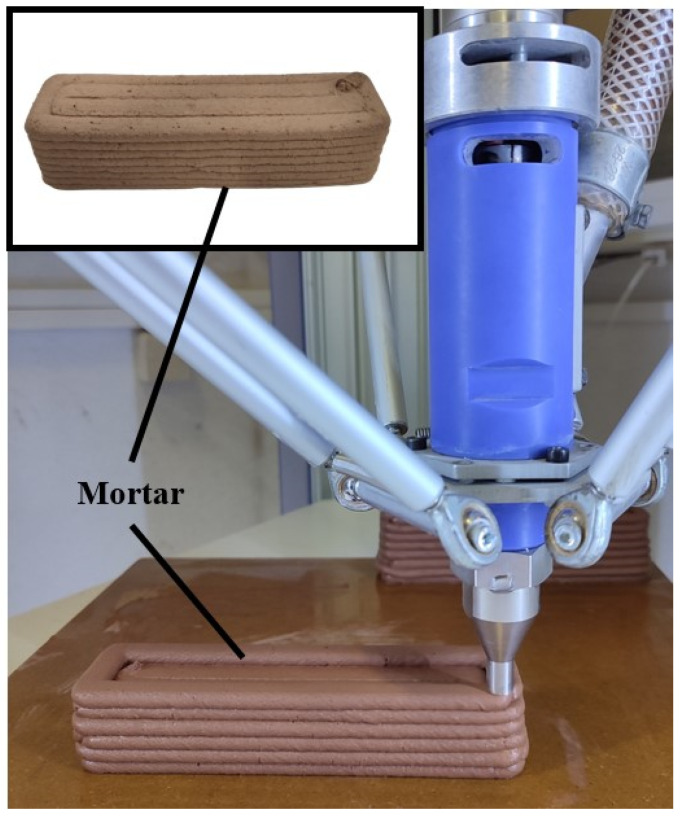
Indicative images of the 3D printing process and the 3D-printed clay-based specimens.

In a previous study by Pemas et al. [41], clay-based prisms were fabricated using liquid deposition modeling (LDM) AM technology and designed with SOLIDWORKS^®^ CAD Software (2022 SP2.0 Professional version). The specimens were printed with the WASP 40100 LDM 3D printer, and parameters were set using Simplify3D (Version 5.1.2) software. The fresh clay-based mixture was produced according to EN1015-11 [42], and the specimens were printed with dimensions of 160 mm × 40 mm × 40 mm each. Specifically, the clay-based mixture consisted of clay as a binder, ceramic powder as an aggregate, water, and a polycarboxylate superplasticizer (Master Glenium 11) supplied by BASF (Ludwigshafen, Germany) to decrease water usage. The printing parameters applied for printing the 160 mm × 40 mm × 40 mm specimens included a nozzle diameter of 10 mm and a layer height of 5 mm. The fill density was set at 100%, and the specimens were printed at room temperature. In total, three prisms made with this mixture were printed and tested according to BS EN1015-11:1999 [43] to determine their flexural and compressive strength. In the present study, the mechanical properties obtained from the aforementioned specimens will be used as input data in the process of developing the FEA models presented in the following sections. Table 1 presents the measurements obtained from the mechanical testing. Based on these results, Finite Element Models were developed in the context of this study in order to evaluate the compressive and flexural behavior of clay-based prisms reinforced with polymeric bioinspired lattice structures. In addition, Figure 2 illustrates indicative images from the 3D printing and the 3D-printed clay-based specimens, and the layers of the structure are visible, showing the region where the corresponding reinforcement structure can be added manually by the users during the additive manufacturing process.

### 2.2. Design Process

**Figure 3:**
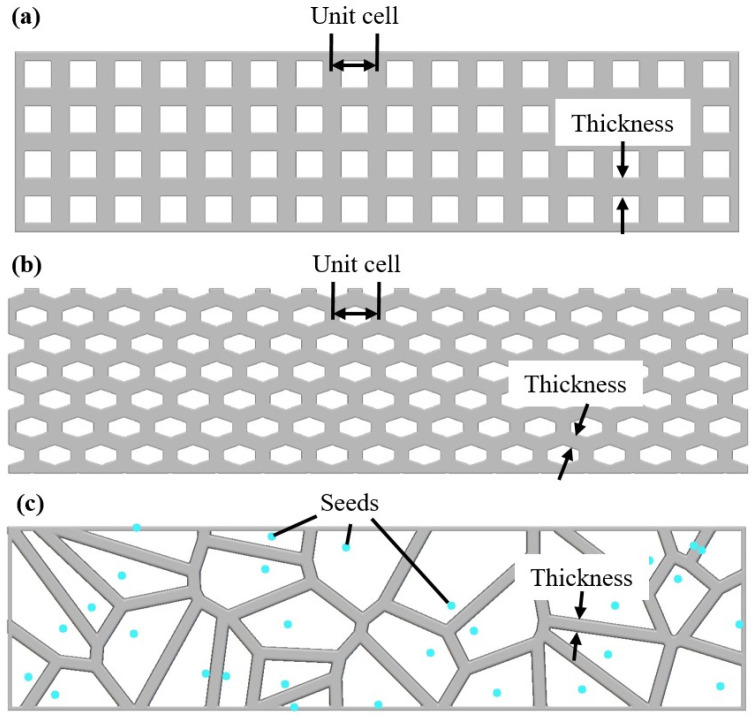
Indicative images of the selected 2.5D architected material along with their main design features: (**a**) Cubic mesh, (**b**) Honeycomb mesh, and (**c**) Voronoi mesh.

In the current study, the aforementioned prisms were redesigned embedding bioinspired lattice structures. These lattice structures are 3D-printed panels consisting of 2.5D architected materials with external dimensions of 160 mm × 40 mm × 2 mm. The developed panels are inserted in the prism structures during the LDM 3D printing process in every layer of the structure (5 mm). It is worth noting that in the actual process, the polymeric panels were immersed in the clay-based paste resulting in an actual clay clearance of 3 mm. In this paper, three entirely different 2.5D architected materials were selected in order to be evaluated for reinforcement of the clay-based prisms [44]. The first architected material was a 2.5D cubic grid, which is the simplest lattice structure yet the most commonly used in civil engineering applications [45]. The second chosen architected material was the 2.5D honeycomb-like lattice structure which is a bioinspired structure well-known for its remarkable mechanical behavior [46]. Finally, for the third architected material, a stochastic lattice structure was employed utilizing the bioinspired Voronoi design algorithm. The use of the Voronoi structure is a novel approach to reinforcement grids that has been known to increase interest in the evolution of additive manufacturing [47]. Figure 3 portrays indicative images of the selected 2.5D architected materials. It is worth noting that these architected materials were developed and designed utilizing nTopology design software (nTopology, New York, NY, USA).

The numerical evaluation of the lattice structure reinforcement was performed in three different relative densities for each selected architected material due to the influence of the size effect [48]. The size effect is the phenomenon that describes how the response of an architected material is degraded by the reduction in the relative density. This effect is intense for architected material below 60% relative density and obeys the following scaling law equations where *Φ_solid_* is the mechanical property of the construction material, *Φ_lattice_* is the effective mechanical property for the lattice structure, and G and *n* are constants whose values depend on the construction material and the applied architected material [49].

(1) $$\frac{\Phi_{lattice}}{\Phi_{solid}}=G\cdot {\left(\bar{\rho }\right)}^{n}$$

The three distinct relative densities for each employed lattice structure were chosen to be 30%, 50%, and 70%. Table 2 lists the values of the main design parameters, namely the strut thickness (t_s_), the unit cell length (l), and the seed distance (l_s_) for each architected material in order to achieve the desired relative densities. In addition, it is worth mentioning that each design panel has a height of 2 mm and the changes in the relative density of each structure were performed by modifying the strut thickness.

### 2.3. Finite Element Models

To numerically evaluate the developed clay-based prism reinforced by the aforementioned bioinspired lattice structures, Finite Element Models (FEMs) were developed, simulating the compressive and flexural response of each specimen. For this purpose, the static module of the ANSYS Workbench simulation platform (ANSYS, Inc., Canonsburg, PA, USA) was used. The first step in this process was the setup of material models. For the developed clay-based prism with polymeric reinforcement, two different material models were utilized, one for clay and one for the polymer. In addition, a bilinear isotropic hardening material model was deployed in order to simulate accurately both the elastic section but also a sufficient portion of the plastic section. For the clay-based material, the mechanical properties of Table 1 were used along with a Poisson ratio of 0.35. Regarding the polymeric lattice structure, the widely used PLA material was selected as construction material. Table 3 lists the main properties of 3D-printed PLA as they were derived from a previous study [50]. It is worth noting that the tangent modulus for both materials was set at a zero value, which is a legitimate assumption for these kinds of materials.

**Figure 4:**
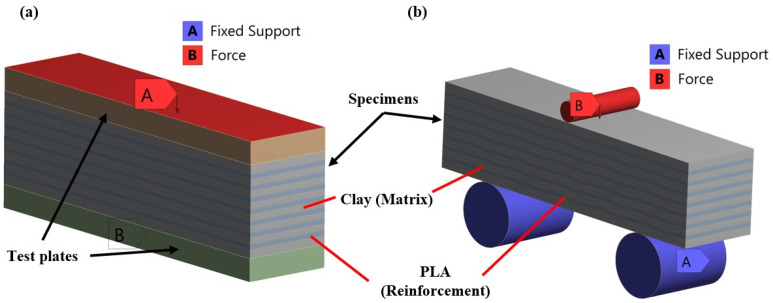
Indicative images of the loading scenarios: (**a**) compression and (**b**) bending testing.

Furthermore, the composite prisms with clay-based and PLA materials were set to have bonded contact regions, a valid assumption due to the bonding properties of the solidified clay. Moreover, a mesh sensitivity analysis was performed to extract the minimum number of elements that could capture accurately the advanced morphology of the examined structure. This analysis was conducted based on the extracted equivalent von Mises stresses. The results of this analysis led to the utilization of tetrahedral elements with a minimum element size of 1 mm resulting in computation meshes between 400,000 and 500,000 elements. Regarding the loading conditions, there were two different loading scenarios, one for compression testing and one for three-point bending testing. Figure 4 graphically shows these two loading scenarios. For the three-point bending, the test configuration of a previous study was used [41] to extract numerical results that will be comparable with future experimental results. This configuration had a length span of 90 mm, with two fixed cylinders of 30 mm diameter on the bottom side and one moving cylinder (probe) of 9.8 mm diameter on the upper side. It is worth mentioning that the execution of these finite element analyses (FEAs) was performed in 10 distinct steps in order to achieve smoother convergence and extract the mechanical response in each of these steps.

## 3. Results

### 3.1. Final Designs

**Figure 5:**
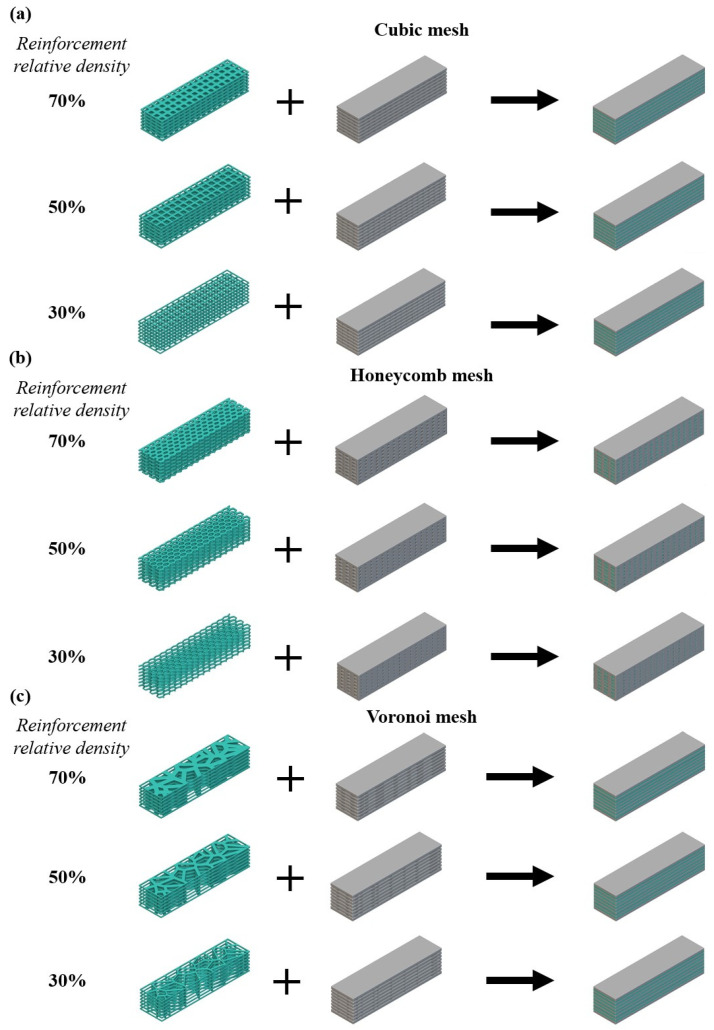
Developed prism designs for: (**a**) cubic mesh, (**b**) honeycomb mesh, and (**c**) Voronoi mesh.

Through the aforementioned design methodology, nine distinct designs of clay-based prisms reinforced with bioinspired lattice structures were produced into three different relative densities for each of the three selected architected materials. Figure 5 presents all the designed prisms highlighting both their matrix and their reinforcement. 

Regarding the as-designed density and weight of each prism, Table 4 presents the corresponding calculated values. In each of these nine different designs of reinforced clay-based prisms, there were seven panels of polymeric lattice reinforcement with a spacing of 3 mm. It is worth mentioning that the first and the last layers of the prism were designed at 4 mm following the result of a physical assembly of the reinforcement.

### 3.2. Numerical Evaluation

Utilizing the above-presented prism designs, FEAs were conducted for both compression and three-point bending testing. Through these FEAs, the compressive and flexural strengths were evaluated for each specimen coupled with the observed elastic modulus. Moreover, exploiting these results, the corresponding scaling laws were calculated, quantifying the mechanical behavior of these composite prisms for a sufficient range of relative density of the reinforcement structure. It is worth noting that for both loading scenarios, a pure clay-based prism was also simulated to use it as a benchmark for the developed structures.

Regarding the compressive behavior of the developed prisms, Table 5 lists the basic mechanical properties as they were derived from compression testing using FEAs. The acquired data showed that the clay-based prisms with the honeycomb reinforcement revealed the highest stiffness and strength, followed by ones with cubic reinforcement and the Voronoi structure. In addition, all examined reinforced prisms exhibited higher stiffness than the pure clay-based, which was expected due to the higher elastic modulus of the PLA material. In terms of strength, both the yield strength and the peak strength followed a similar trend, highlighting the honeycomb reinforcement as the best structure for this purpose, reaching a peak compressive force of around 17,000 N. Again, the reinforced prisms showed higher strength in general than the pure clay-based prisms. These results occur due to the high connectivity and more uniform stress distribution that are provided by the lattice structure reinforcement. The superior performance of the honeycomb reinforcement structure can be attributed to its morphology, which offers an exceptional strength-to-weight ratio, enhanced load distribution, and increased resistance to deformation and stress concentration. This geometry enables the honeycomb structure to more effectively and more uniformly bear and distribute applied forces compared to the other structures analyzed in this study.

**Figure 6:**
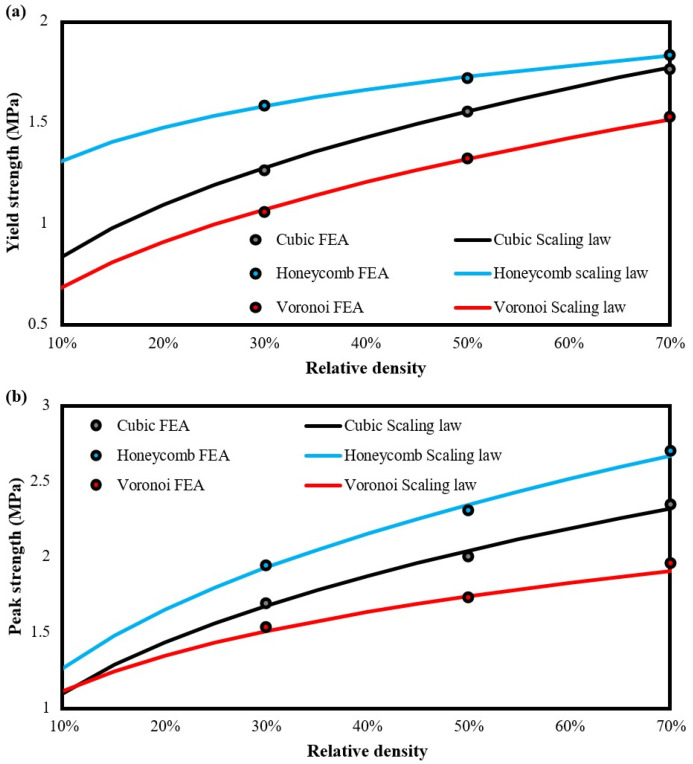
Curve-fitted scaling laws of all examined reinforcement structures for: (**a**) yield strength and (**b**) peak strength.

The next step was the evaluation of the constants of the corresponding strength scaling law for the examined prisms. The scaling law parameters were calculated by applying a curve-fitted process for each corresponding power law. Table 6 presents the values of C and *n* for each employed lattice structure. Furthermore, Figure 6 portrays the scaling law curves for yield strength and peak strength, respectively. Through these formulations, a numerical tool is provided for each examined structure with which the strength of the developed composite can be easily evaluated for a wide range of relative density, i.e., from 10% to 70% of the applied reinforcement structure.

It is worth noting that below 10% relative density the reinforcement structure reveals brittle behavior leading to the specimen’s failure. On the other hand, above the 70% relative density, a block of polymeric material is created, resulting in discrete regions of different materials. Regarding the trend of scaling laws, the observed behavior was an intense stretching-dominated behavior [44], which was expected, due to the function of clay-based material as the bonding agent between the reinforcement structure, providing that way increasing connectivity between the elements of each structure. Finally, the curve-fitting process seems to be relatively accurate and reliable, achieving a coefficient of determination (R^2^) above 0.995.

Similar findings were observed in the bending testing via FEAs where reinforced clay-based prisms exhibited higher flexural strength, i.e., maximum force, than the pure clay-based prisms, as it is presented in Table 7. Again, honeycomb reinforcement revealed the highest flexural strength at 3.41 MPa, reaching a maximum bending strength of 1553 N. On the other hand, cubic and Voronoi reinforcement has similar strengths with cubic structure showing a little higher flexural strength in general. 

**Figure 7:**
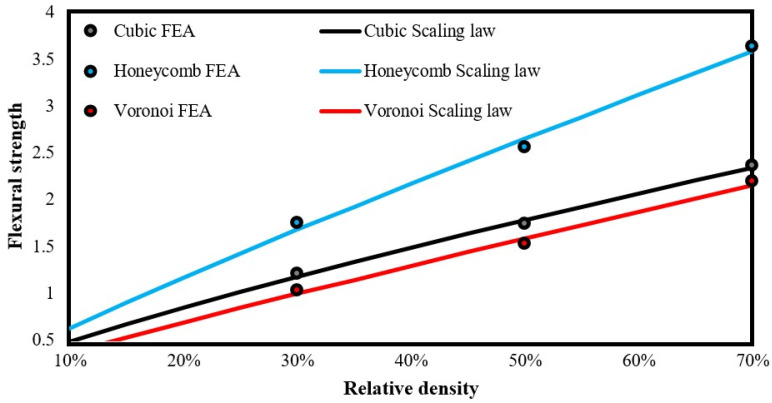
Curve-fitted scaling laws of all examined reinforcement structures for flexural strength.

In addition, in the bottom part of Table 7, the quantified constant of the scaling law for the flexural strength of each developed reinforced prism is depicted. Moreover, Figure 7 illustrates the corresponding curve-fitted scaling laws. These curves follow an almost linear trend highlighting the increased connectivity of the prism’s elements due to the existence of reinforcement structure. Again, it is obvious that the honeycomb reinforcement offers the best performance in terms of flexural strength with a substantial difference from the other reinforcements. It is also worth mentioning that the reliability and accuracy of the below-presented curve-fitted process reaches values of R^2^ above 0.995.

**Figure 8:**
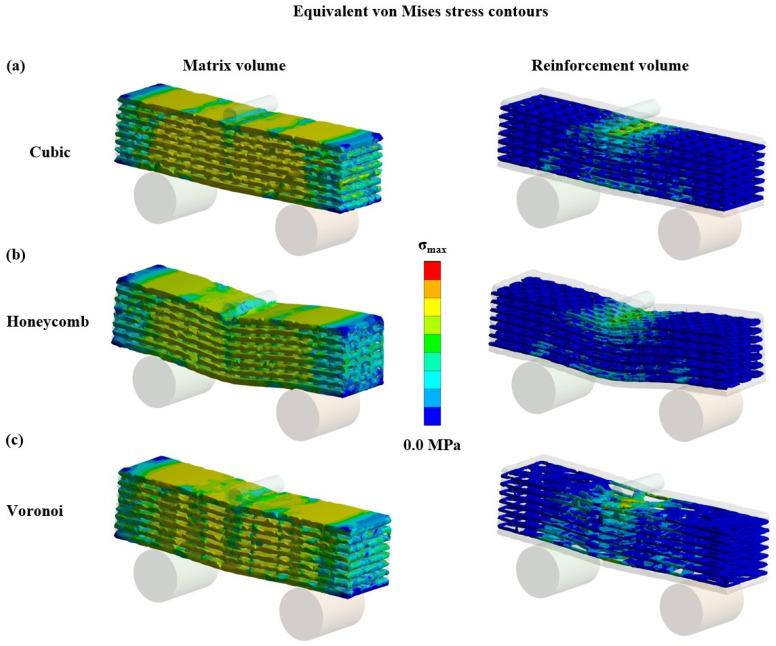
Equivalent von Mises stress contours of 70% reinforced specimens for: (**a**) cubic, (**b**) honeycomb, and (**c**) Voronoi prisms.

Finally, in Figure 8, indicative images of FEA results are depicted focusing separately on the two elements of the developed prism, namely the matrix and the reinforcement. More specifically, Figure 8 shows the equivalent von Mises stress distribution during the bending loading.

As it was expected, the matrix disturbed the stress uniformly within the body of the entire structure. On the other hand, the reinforcement revealed a behavior, which is similar to the corresponding behavior of reinforced cement structures. In detail, the reinforcement layer near the load application point experiences compressive loads in contrast with the layer close to the fixation, which exhibited tensile loads due to the developed elastic deflection. This observed behavior is the one that led to an enhanced flexural response for the reinforced prism specimens.

## 4. Conclusions

In this study, a systemic approach incorporating computer aid and biomimetics is utilized for the development of 3D-printed clay-based composite mortar reinforced with advanced polymeric bioinspired lattice structures, namely, cubic grid, honeycombs, and Voronoi architected materials. The designed specimens were numerically examined under compression and bending loading and the corresponding mechanical performance was evaluated. Moreover, three different relative densities of reinforcement were employed in order to quantify the scaling laws for these complex structures, providing essential mathematical formulations to predict the mechanical response of a composite depending on the applied relative density. Through this series of computer-aided numerical analyses, the honeycomb reinforcement was highlighted as the one with the best mechanical performance in both compression and bending, followed by the cubic grid and the Voronoi structures. More specifically, honeycomb reinforcement at 70% relative density almost doubled the peak compression strength and tripled the flexural strength of the overall structure. These findings indicate that clay-based composite mortar reinforced with bio-based polymeric honeycomb, such as the PLA constructed, is capable of withstanding increased loads. Therefore, in the future, this type of structure can be further examined through experimental testing in order to verify its exceptional mechanical performance and pave the way for its integration into real-life applications. The ultimate objective of this study is to identify a more environmentally sustainable material that can potentially replace cement in construction, which is accompanied by a significant amount of CO2 emissions.

## Figures and Tables

**Table 1:** Compressive and flexural mechanical properties.

Specimens	Density	Elastic Modulus	Compressive Strength	Flexural Strength
Clay-based mixture	1.73 g/cm^3^	4710 MPa	1.57 MPa	1.01 MPa

**Table 2:** Design parameters for the lattice structures.

Relative Density	Cubic		Honeycomb		Voronoi	
	t_s_ (mm)	l (mm)	t_s_ (mm)	l (mm)	t_s_ (mm)	l_s_ (mm)
30%	1.55	10	1.20	10	2.30	6
50%	2.75		2.10		4.20	
70%	4.20		3.25		6.5	

**Table 3:** Main properties of 3D-printed PLA material [50].

Properties	Values
Density	1.23 g/cm^3^
Poisson ratio	0.35
Elastic modulus	3000 MPa
Peak strength	63 MPa
Elongation at break	20%

**Table 4:** Weight and density of each designed prism specimen.

Specimens	Weight	Density
Pure Clay-based	442.88 g	1.73 g/cm^3^
Clay-based with 30% PLA reinforcement	430.23 g	1.68 g/cm^3^
Clay-based with 50% PLA reinforcement	422.08 g	1.65 g/cm^3^
Clay-based with 70% PLA reinforcement	413.48 g	1.62 g/cm^3^

**Table 5:** Basic mechanical properties from compression testing.

Specimens	Elastic Modulus	Yield Strength	Peak Strength
**No reinforcement**			
Pure clay-based	4710 MPa	1.12 MPa	1.57 MPa
**Cubic mesh**			
Clay-based with 30% PLA reinforcement	4100 MPa	1.26 MPa	1.69 MPa
Clay-based with 50% PLA reinforcement	3648 MPa	1.55 MPa	2.01 MPa
Clay-based with 70% PLA reinforcement	3010 MPa	1.76 MPa	2.34 MPa
**Honeycomb mesh**			
Clay-based with 30% PLA reinforcement	4020 MPa	1.58 MPa	1.94 MPa
Clay-based with 50% PLA reinforcement	3880 MPa	1.72 MPa	2.32 MPa
Clay-based with 70% PLA reinforcement	3650 MPa	1.83 MPa	2.69 MPa
**Voronoi mesh**			
Clay-based with 30% PLA reinforcement	4130 MPa	1.05 MPa	1.53 MPa
Clay-based with 50% PLA reinforcement	3530 MPa	1.32 MPa	1.67 MPa
Clay-based with 70% PLA reinforcement	3140 MPa	1.56 MPa	1.95 MPa

**Table 6:** Scaling law parameters for the employed reinforcement structures.

Type of Reinforcement	Scaling Law Parameters			
	Yield Strength		Peak Strength	
	C_y_	n_y_	C_p_	n_p_
Cubic mesh	1.815	0.387	1.687	0.383
Honeycomb mesh	1.734	0.172	1.939	0.383
Voronoi mesh	1.562	0.409	1.336	0.277

**Table 7:** Basic mechanical properties from bending testing.

Specimens	Maximum Force	Flexural Strength
**No reinforcement**		
Pure clay-based	431 N	1.01 MPa
**Cubic mesh**		
Clay-based with 30% PLA reinforcement	517 N	1.21 MPa
Clay-based with 50% PLA reinforcement	746 N	1.75 MPa
Clay-based with 70% PLA reinforcement	1010 N	2.37 MPa
**Honeycomb mesh**		
Clay-based with 30% PLA reinforcement	751 N	1.76 MPa
Clay-based with 50% PLA reinforcement	1094 N	2.56 MPa
Clay-based with 70% PLA reinforcement	1553 N	3.41 MPa
**Voronoi mesh**		
Clay-based with 30% PLA reinforcement	440 N	1.03 MPa
Clay-based with 50% PLA reinforcement	654 N	1.53 MPa
Clay-based with 70% PLA reinforcement	937 N	2.19 MPa
**Type of reinforcement**	**Scaling law parameters**	
	**Flexural strength**	
	**C_b_**	**n_B_**
Cubic mesh	3.099	0.811
Honeycomb mesh	4.881	0.894
Voronoi mesh	2.947	0.909

## Data Availability

The raw data supporting the conclusions of this article will be made available by the authors on request.
